# Congenital Defects in Neutrophil Dynamics

**DOI:** 10.1155/2014/303782

**Published:** 2014-08-05

**Authors:** Marton Keszei, Lisa S. Westerberg

**Affiliations:** Department of Microbiology Tumor and Cell Biology, Karolinska Institutet, 171 77 Stockholm, Sweden

## Abstract

Neutrophil granulocytes are key effector cells of the vertebrate immune system. They represent 50–70% of the leukocytes in the human blood and their loss by disease or drug side effect causes devastating bacterial infections. Their high turnover rate, their fine-tuned killing machinery, and their arsenal of toxic vesicles leave them particularly vulnerable to various genetic deficiencies. The aim of this review is to highlight those congenital immunodeficiencies which impede the dynamics of neutrophils, such as migration, cytoskeletal rearrangements, vesicular trafficking, and secretion.

## 1. Introduction

Congenital immunodeficiencies related to neutropenia or neutrophil dysfunction account for 10–20% of primary immunodeficiencies [1, 2]. These diseases are characterized by severe recurrent bacterial and fungal infections which often affect the respiratory tract, skin, and oral cavity and sometimes manifest at unusual sites such as brain or liver abscesses.

**Figure 1:**
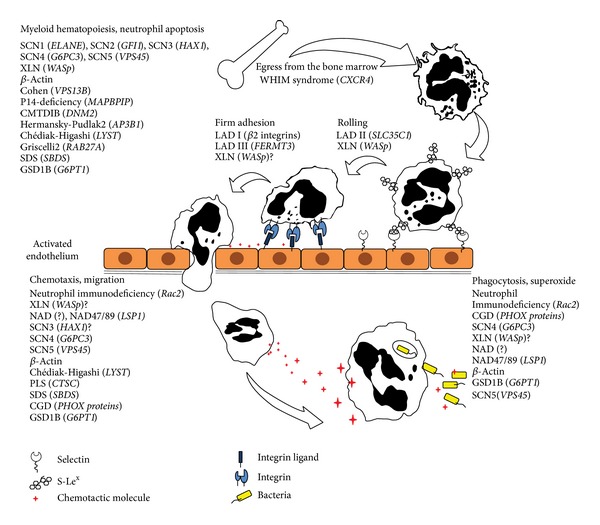
Neutrophil development, migration, and function. Diseases described in the review are indicated where they are believed to act.

Neutrophils are first responders to bacterial infections. They follow various chemotactic gradients and they are recruited in large numbers from blood through the endothelium to the infected tissue where they release vesicles loaded with proteolytic enzymes and antimicrobial peptides (Figure 1). Upon encountering bacteria neutrophils capture, ingest, and kill them by production of reactive oxygen species. Abnormalities in any aspects of neutrophil development and/or function induce immunodeficiency or aberrant inflammatory reactions (Table 1) which reflects in the complexity of the diagnosis of these diseases [2]. A common denominator in these diseases is failure to properly regulate the actin cytoskeleton by direct or indirect genetic mutations. Such failure is implicated in decreased migratory and adhesive properties, altered vesicle dynamics and release, and perturbed assembly of the NADPH oxidase necessary for antimicrobial killing by neutrophils. Here we propose that the failure to regulate the actin cytoskeleton and vesicle trafficking is a unifying component in many neutrophil deficiencies.

## 2. Defects of the Actin Cytoskeleton and Cell Adhesion

Actin is a globular protein which binds ATP (or ADP) and can be found in all eukaryotic cells. Actin polymerization in the cell cortex plays a fundamental role in cell motility. Polymerized actin forms a leading edge, a membrane protrusion in cells that creates sufficient forces to propel cell movement. These propelling forces in molecular scale originate from rapid assembly and disassembly of globular G-actin monomers to filamentous F-actin polymers [3]. Spontaneous nucleation of actin filaments is slow since, unlike the polymer which is stabilized by contacts between several subunits, dimers and trimmers are unstable. Cells control new filament assembly through the induction of nucleation promoting factors such as the WASp/WAVE (Wiskott-Aldrich syndrome protein/WASp-family verprolin-homologous protein) family proteins. These factors stimulate the Arp2/3 protein complex to nucleate actin polymerization in the side of an existing polymer as a branch. New filaments grow rapidly, in a rate limited by the concentration of available actin monomers, and they push the plasma membrane forward. This transient growth is terminated by the binding of capping proteins to the fast-growing (barbed) end of the filament. Breakage of filaments is catalyzed by actin-severing proteins such as gelsolins and the ADF/cofilin family. Severed filaments shorten and debranch. As a result of the action of nucleation promoting factors, capping and actin severing proteins, and several other actin binding accessory proteins, F-actin forms a tightly regulated 3-dimensional network which is growing in the leading edge and disassembles some distance in the rear thereby creating a plasma membrane protrusion [3].

**Figure 2:**
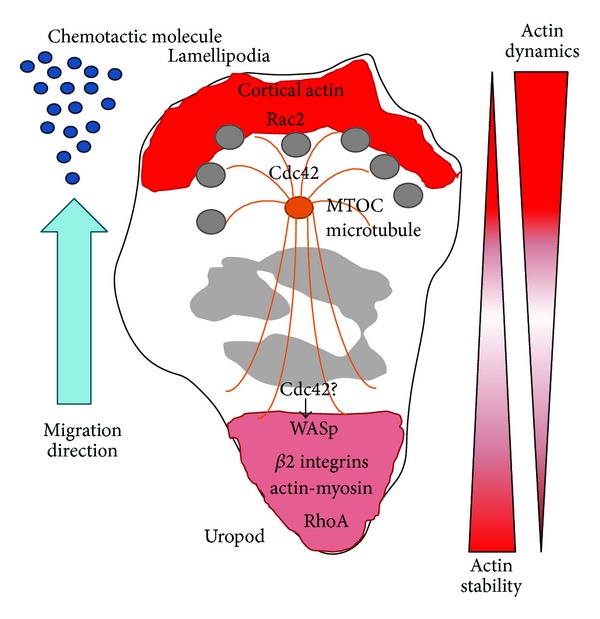
Neutrophil polarity during migration. The role of the cell cytoskeleton and the proteins that regulate cell polarity is indicated.

Neutrophils polarize their cytoskeleton to form a leading edge (lamellipodia or pseudopod) towards the signal of origin and a trailing uropod in the posterior of the cell. While the leading edge consists of highly branched and dynamic actin filaments, the uropod is rich in actin-myosin II contractile structure. During chemotaxis, the cells extend the leading edge by local actin polymerization and contract the uropod to allow movement in the direction of the signal. It has become increasingly clear that the Rho GTPases Cdc42, Rac, and Rho serve a key role in establishment of cell polarity. By direct binding to the WASp family of proteins they regulate localized actin polymerization and interaction with cell surface chemokine receptors and integrins [4] (Figure 2).

### 2.1. *β*-Actin Dysfunctions

Actin proteins are highly conserved evolutionary in vertebrates and their functional integrity is essential for the survival of a complex organism. Out of the six actin isoforms, the nonmuscle *β*-actin is ubiquitously expressed in all cell types and the deletion of this isoform is embryonic lethal in mice [5, 6]. A single case study of a patient carrying a heterozygous *β*-actin E364K mutation reported recurrent infections, thrombocytopenia, photosensitivity, and mental retardation [7]. The patient exhibited profound neutrophil functional defects in chemotaxis, superoxide production, and membrane potential response. These defects were attributed to impaired binding of the E364K *β*-actin to the actin-binding protein, profilin. Another mutation in *β*-actin, R183W, causes malformations, deafness, and neurological abnormalities such as dystonia [8]. Yet another set of mutations in *β*-actin have been recently identified to cause Baraitser-Winter syndrome (BRWS). BRWS is a rare condition, characterized by ocular colobomata, ptosis, neuronal migration defect, distinct craniofacial anomalies, and intellectual disability [9–11]. Remarkably, the neutrophil dysfunction (*β*-actin E364K), dystonia (*β*-actin R183W) [8], and BRWS [7, 9] cases were presumably caused by dominant missense mutations in *β*-actin. Although no immunological defects were reported either in the *β*-actin R183W case or BRWS cases, both reports found abnormal F-actin structures in mutant *β*-actin transfected cell lines [8, 9]. The BRWS associated R196H mutation induces greatly increased F-actin with multiple, anomalous F-actin-rich, filopodia-like protrusions compared to control cells in lymphoblastoid cell lines [9]. Both the BRWS mutation R196H and the dystonia mutation R183W mutation render F-actin more resistant to the depolymerizing effect of Latrunculin A in lymphoblasts. These results suggest that accumulation of filamentous actin plays an important role in diseases caused by mutations in *β*-actin. While there is yet no evidence that the R183W and BRWS mutations in *β*-actin affect the immune system broadly, given the neutrophil dysfunction in the E364K patient together with the central role and abundance of *β*-actin in leukocytes, we reason that neutrophil function is likely to be compromised.

### 2.2. WASp Deficiency and Overactivity

Patients with Wiskott-Aldrich syndrome (WAS) lack or have reduced expression of WASp and suffer from combined immunodeficiency with recurrent infections [12, 13]. WASp is uniquely expressed in hematopoietic cells and resides as an inactive form in the cytoplasm due to an autoinhibited folding where its GTPase binding domain forms a molecular interaction with the carboxy-terminal verprolin-cofilin homology and acidic (VCA) domain. Upon signaling, the small Rho GTPase Cdc42 binds to WASp that undergoes a conformational change to open up the protein. This exposes the carboxy-terminal part of the protein that binds directly to the Arp2/3 complex and induces actin polymerization. It may not be surprising that neutrophils lacking WASp have defects in all responses that depend on the actin cytoskeleton such as F-actin polymerization, migration, adhesion under flow, and *β*2-integrin clustering [14, 15]. WASp^−/−^ neutrophils exhibit multiple F-actin fronts and fail to redistribute CD11b into clusters at the uropod [14, 16]. A recent report shows that in neutrophils, WASp seems to be dispensable for F-actin polymerization at the leading edge [16]. Instead, Cdc42 activates WASp at the uropod and facilitates microtubule capture and stability at the uropod via clustering of CD11b *β*2 integrins [16].

The more recently described X-linked neutropenia (XLN) is caused by mutations (L270P, S272P, I276S, and I294T) in the GTPase binding domain of WASp and destroys the autoinhibited conformation of WASp [12, 13]. These mutations were initially predicted to lead to constitutively active WASp and as a consequence cells would have increased load of polymerized actin [17]. Several laboratories have now confirmed this hypothesis and shown markedly increased polymerized actin in neutrophils, in macrophages, and in B and T cells [18–22] (Keszei and Westerberg-unpublished observation). XLN patients suffer from recurrent bacterial infections because of severe neutropenia and monocytopenia [17, 18, 20] and they may develop cytogenetic changes indicative of chromosomal instability, myelodysplasia, or acute myeloid leukemia [18–20, 22]. Neutrophils from XLN patients have decreased capacity to phagocytose bacteria and kill them [18]. Oxidative burst in XLN neutrophils is normal in response to PMA, while receptor-mediated oxidative burst in response to* E. coli *or fMLP is reduced [18]. This suggests that XLN neutrophils fail to effectively assemble signaling complexes at the cell membrane. One recent report shows that excess cytoplasmic F-actin in XLN causes increased cellular viscosity and tension and this indirectly perturbed mitotic mechanics [23]. Membrane tension appears to be one mode of long-range inhibition mechanisms. Membrane tension nearly doubles during leading edge protrusions, and increase in tension is sufficient for long-range inhibition of Rac activation at the leading edge [24]. In contrast, reduced membrane tension activates actin assembly throughout the cell [24]. Macrophages from XLN patients have increased turnover rate of actin-rich adhesive structures called podosomes [18] and murine XLN B and T cells can adhere to antibody-coated layers but fail to coordinate cell spreading [22]. B cells from XLN patients form less dynamic contacts with L-selectin ligands under flow [21]. This is likely to be caused by excessive localized production of cortical F-actin that induces increased rigidity of microvilli [21]. Neutrophils devoid of Rac2 (discussed below) are also unable to adhere to L-selectin ligand under flow despite normal levels of L-selectin expression [25]. Together this highlights the importance for dynamic cytoskeletal rearrangement in L-selectin-dependent rolling on endothelial cells. How increased load of polymerized actin in XLN affects cell polarity, migration, and tension in neutrophils remains to be determined.

### 2.3. Neutrophil Immunodeficiency Syndrome (Rac2)

Rac2 belong to the Rho family of small GTPases that act as molecular switches inside the cell by cycling between a GDP-bound inactive form and a GTP-bound active form [26]. The activity of Rho GTPases also depends on their localization to lipid membranes by posttranslational addition of lipid anchors. In neutrophils, Rac2 is highly polarized to the leading edge where it regulates actin assembly by activating the WASp family members. Another Rho GTPase, RhoA, is localized to the trailing uropod where it coordinates actin-myosin filaments. A third Rho GTPase member, Cdc42, is a key regulator of cell polarity by assembly of the microtubule organizing center (MTOC) between the leading edge and the cell nucleus. Rac2 is highly expressed in neutrophils and is essential to assembly of the NADPH oxidase that initiates production of toxic oxygen metabolites to kill pathogens [27]. Three patients with mutations in Rac2 have been identified that suffer from a neutrophil immunodeficiency syndrome. Curiously, all three patients harbor a D57N mutation within the DX_2_G motif, conserved in all GTPases, that results in a dominant negative protein. Rac2-D57N neutrophils show complete loss of chemotaxis, azurophil granule secretion, superoxide generation, and polarization in response to a variety of receptor stimuli, especially the chemokine fMLP [28–30]. Murine Rac2^−/−^ neutrophils show a similar phenotype and have perturbed polarization and decreased capacity to migrate in vitro and in vivo into the peritoneum [25]. Moreover, Rac2^−/−^ neutrophil have decreased NADPH function associated with reduced clearance of the opportunistic pathogen* A. fumigatus.*

The critical role of NADPH activity for neutrophil function is highlighted in chronic granulomatous disease (CGD), characterized by severe, life-threatening bacterial and fungal infections and immune dysregulation [31]. CGD is caused by mutation in any one of the five subunits of the NADPH oxidase, including gp91phox (cytochrome b-245, *β*-polypeptide, CYBB), p22phox (cytochrome b-245, *α*-polypeptide, CYBA), p47phox (neutrophil cytosolic factor 1, NCF1), p67phox (NCF2), and p40phox (NCF4). CGD patients have defective microbial activity resulting from abolished superoxide production. Studies of CGD patients neutrophils suggest that assembly of the NADPH complex is not only important for oxidative killing of microbes. The microbial spectrum of infections in CGD includes bacteria that require neutral pH for effective nonoxidative killing and are resistant at the acid pH found in the phagosomes of CGD neutrophils. These include* S. aureus, S. marcescens, N. asteroids,* and* A. fumigatus*. This implies that reactive oxygen species produced by the NADPH oxidase also act as intracellular signalling molecules, leading to the activation of other nonoxidative pathways for microbial killing. One possible mechanism whereby reactive oxygen species could contribute to lamellipodia and thereby increased motility of neutrophils is through cofilin. Reactive oxygen species induce cofilin dephosphorylation through activation of the cofilin phosphatase Slingshot [32]. When dephosphorylated, cofilin binds existing cortical actin filaments and severs them. This generates new barbed ends on the filaments to which the Arp2/3 complex can bind and stimulate branching and thereby increase dynamics of the lamellipodia [33]. One implication is that, in the absence of NADPH oxidase activity, neutrophils have less capacity to form a dynamic lamellipodia required for migration [34] and that phagocyte enzymes are present but hypofunctional [35].

### 2.4. Neutrophil Actin Dysfunction (NAD) Syndrome

One case of neutrophil actin dysfunction (NAD) was reported in 1974 in a male newborn patient [36]. The patient had recurrent bacterial infections despite marked neutrophilic leukocytosis, impaired neutrophil migration from blood to the inflammation site, and impaired phagocytosis by neutrophils. The patient's neutrophils extended a few fork-like pseudopodia and actin isolated from his neutrophils polymerized poorly in vitro. F-actin content in the neutrophils of the patient's father, mother, and sister was significantly lower than in controls [37]. Expression of CR3 subunits (CD11b, CD18) was depressed in the patient's mother and a sister, which argues that NAD is a form of leukocyte adhesion deficiency (LAD, discussed below); however, F-actin content is normal in LAD patients [38]. It had been speculated that NAD is a result of a defect in an actin associated protein; however the gene mutation which caused NAD in the index patient had not been found.

Defective actin polymerization was also found in a 2-month-old male infant with recurrent fevers and fungal infections [39]. The neutrophils of the patient had frequent development of F-actin rich filamentous projections that were not present in control PMNs and showed profound defect in random migration, chemotaxis toward fMLP, and phagocytosis. In this patient, CD11b expression was increased. In contrast to the other NAD case, cell lysates from this patient showed a significant decrease in an 89 kDa protein and a marked increase in a 47 kDa protein. Coates and colleagues named this disease actin dysfunction NAD 47/89. The overexpressed 47 kDa protein has been shown to bind actin and its cloning revealed that it was a known actin regulator, lymphocyte-specific protein 1 (LSP1) [40], which is expressed in normal neutrophils [41]. LSP1 overexpression produces F-actin bundles and hair-like surface projections in several eukaryotic cell lines. Moreover, increased expression of LSP1 inhibits the locomotion of normally motile human melanoma cells [42]. On the other hand, murine neutrophils devoid of LSP1 expression have increased migratory capacity. Together these data show that LSP1 is a negative regulator of neutrophil chemotaxis [43].

### 2.5. Leukocyte Adhesion Deficiency (LAD)

During the course of an infection neutrophils leave the blood stream in large numbers by transmigrating the endothelium. The complex process of transmigration is tightly regulated in order to segregate the homeostatic tissue environment from blood vessels which carry a large number of potentially damaging leukocytes. Local inflammation quickly activates the adjacent endothelium which upregulates P- and E-selectins that binds to sialyl-Lewis^X^ carbohydrates on the neutrophil surface. Swiftly moving neutrophils in blood vessels get tethered to the endothelial surface by selectins and they start rolling on that surface. Chemoattractants, such as CXCL8 (IL-8), activate *β*2 integrins on neutrophils which in turn bind intercellular adhesion molecule-1 and molecule-2 (ICAM-1, ICAM-2) on the activated endothelium and mediate firm adhesion between neutrophils and the endothelium. This firm adhesion is prerequisite for extravasation. Aberrations in these processes in LAD patients lead to recurrent skin infections and soft tissue abscesses, periodontal disease, and impaired pus formation despite blood neutrophilia [44]. While LAD II is a result of mutations in a membrane transporter of fucose which impairs selectin mediated adhesion, LAD I is caused by a genetic defect in CD18 (ITGB2). CD18 is a common *β* chain of four *β*_2_ integrins in leukocytes, each containing a different *α* chain: LFA-1 (*α*_L_ *β*_2_ or CD11a : CD18), Mac-1 (*α*_M_ *β*_2_ or CD11b : CD18 which is complement receptor CR3), gp150/95 (*α*_X_ *β*_2_ or CD11c : CD18 which is complement receptor CR4), and ADB2 (*α*_D_ *β*_2_ or CD11d : CD18). Mutations in CD18 fully or partially abolish the expression of *β*_2_ integrins on leukocyte surface, thereby largely impeding neutrophil transmigration into inflamed tissues and renders neutrophils unresponsive to bacteria opsonized with complement fragment C3bi. In contrast, LAD III patients show normal expression of *β*_2_ integrins. Due to mutations in the intracellular protein kindlin-3 (FERMT3) which regulates inside-out integrin activation, the integrins fail to change their conformation to become functionally active.

Integrins clearly depend on the connection to the actin cytoskeleton to carry out their functions [45–47]. They bind to several F-actin associated proteins (talin, vinculin, and *α*-actinin) [46]. Besides anchoring themselves to the actin cytoskeleton, integrins are also involved in induction of local actin polymerization where they engage their ligands on the extracellular matrix on other cells [46]. Intriguingly, it has been shown in knock-out mouse studies that CD11b clustering is abrogated in WASp and Cdc42 deficient neutrophils [16] and the Cdc42/WASp axis acts upstream of integrin functions. These studies suggest that WASp might regulate inside-out integrin signaling in neutrophils and it is critical to maintain neutrophil polarity during migration [16].

### 2.6. Hax1 Deficiency

Approximately 15% of severe congenital neutropenias (SCNs) are caused by autosomal recessive mutations in the* HAX1* gene [48, 49]. Patients with HAX1 mutations present marked neutropenia (absolute neutrophil count < 500 *μ*L^−1^) which causes life-threatening bacterial infections in newborns. HAX1 is involved in B-cell receptor signaling [50] and it has been shown to regulate apoptosis [51, 52]. Neutrophils from HAX1-deficient patients showed higher rate of spontaneous and TNF*α* induced apoptosis than control neutrophils due to loss of mitochondrial membrane potential. It has been suggested that HAX1 is a major inhibitor of apoptosis in myeloid cells and that neutropenia in HAX1-deficient SCN patients is caused by lack of this antiapoptotic function [49]. HAX1 has been shown to interact directly with adhesion and cytoskeleton regulating proteins, such as the actin nucleation-promoting factors cortactin [53] and its homolog hematopoietic lineage cell-specific protein 1 (HS1) [50], *β*6 integrin [54], and G*α*13 [55]. Cavnar and colleagues demonstrated that Hax1 predominantly localize in the leading edge in the PLB-985 neutrophil-like cell line [56]. Knock-down of HAX1 expression results in impaired motility and elongated uropods, as well as decreased RhoA activity. Impaired uropod detachment in HAX1-deficient neutrophils is caused by increased integrin mediated adhesion similarly to neutrophils devoid of RhoA expression. The authors suggest that HAX1 is a negative regulator of integrin-mediated adhesion in neutrophils by affecting Rho GTPase signaling [56].

### 2.7. WHIM Syndrome

Warts, hypogammaglobulinemia, infections, and myelokathexis (WHIM) is an immunodeficiency with autosomal dominant inheritance. In most kindred gain of function mutations of the chemokine receptor CXCR4 have been identified as the cause of the disease [57]. CXCR4 on neutrophils and its ligand, stromal cell-derived factor 1 (SDF1; also known as CXCL12) in the bone marrow stroma, are major bone marrow retention factors for neutrophils [58, 59]. According to a current hypothesis, increased CXCR4-mediated retention signals in bone marrow lead to myelokathexis (hyperplasia with an accumulation of apoptotic neutrophils in the bone marrow) and neutropenia in the periphery [60].

Various early stop codon mutations in WHIM patients have been identified to cause C-terminal intracellular truncations in the CXCR4 protein [57, 61]. Accumulating evidence shows that C-terminal truncations in CXCR4 impair ligand-induced desensitization and internalization of CXCR4. Thereby, an important physiological negative feedback mechanism is interrupted in which CXCR4 activity is downregulated to release neutrophils from the bone marrow [60–63]. Intriguingly, WHIM transgenic zebrafish neutrophils show prominent random membrane protrusions but impaired persistent motility in vivo which resulted in neutrophil retention within areas of high SDF1*α* expression.

## 3. Defects of Vesicular Transport

Neutrophils kill microbes by controlled release of microbicidal products from their secretory granules to the extracellular space and by elimination in neutrophil phagosomes. Neutrophils contain four types of secretory organelles: primary (azurophil) granules, secondary (specific) granules, tertiary (gelatinase) granules, and secretory vesicles. Out of the four organelles, secretory vesicles are mobilized readily, probably already during neutrophil rolling on activated endothelia, and they carry membrane associated proteins such as the *β*_2_ integrin component CD11b to the plasma membrane. This process is thought to transform circulating neutrophils into a highly responsive cell, primed for migration [64]. Gelatinase granules and specific granules are mobilized next and they carry, among other effectors, gelatinase and lactoferrin, respectively. Azurophil granules need the strongest stimulus for their release and they mainly contain myeloperoxidase (MPO), defensins, and neutrophil elastase (NE). Regulated secretion of granules in neutrophils is a complex process which requires sorting of the proteins to this pathways, guiding transport vesicles specifically to secretory granules and mediating membrane fusion and fission. Moreover, vesicle trafficking critically relies on the interplay between the microtubule and actin cytoskeleton. Among others, the small GTPase Cdc42 has the capacity to link these two molecular motor systems to maintain cell polarity. Cdc42 coordinates the microtubule cytoskeleton by binding to the Cdc42 interacting protein (CIP4) that directly regulates microtubule assembly and induces membrane deformation [65]. Cdc42 also coordinates actin polymerization via the activation of WASp and its relative the neuronal (N)-WASp that upon Cdc42 binding becomes active and induces actin polymerization via the Arp2/3 complex [66, 67]. In this way, Cdc42 can mediate the interaction between actin and microtubules and regulate vesicle trafficking. Since neutrophils are packed with potentially harmful substances in granules, correct sorting and release of vesicles is key for neutrophil survival and function. It is reasonable to predict that any change in vesicle trafficking or localization of vesicle components would be harmful for the neutrophil.

### 3.1. Neutropenias with Hypopigmentation

The function of neutrophils, cytotoxic T lymphocytes, natural killer cells, and mast cells is highly dependent on intact secretory machinery for the capacity of these cells to degranulate and release vesicular content towards pathogens and target cells. Genetic defects in degranulation often coincide with impaired melanin secretion by melanocytes indicating the usage of similar secretory pathways [68].

Chédiak-Higashi syndrome (CHS) is characterized by immunodeficiency, hypopigmentation, and neurologic symptoms [69]. Patients develop recurrent pyogenic infections and often periodontal disease which is associated with neutropenia [70], impaired neutrophil chemotaxis [71], and reduced bactericidal activity [72]. A key feature of CHS is the presence of giant granules in most nucleated cells due to aberrant vesicle fusion or fission. Neutrophil granules are deficient in cathepsin G and NE [73] and mobilization of the giant granules is impaired in CHS patients [74]. In fact, enlarged granules might impair cell kinetics mechanically [71]. Mutations in* LYST*, a lysosomal trafficking regulator gene, have been identified as the cause of CHS [75].

Griscelli syndrome type 2 is characterized by partial albinism and marked immunodeficiency including frequent pyogenic infections associated with neutropenia [76]. Mutations in the small GTPase* RAB27A* gene were identified as the cause of disease [77]. The Rab family of GTPases control trafficking of vesicles between intracellular compartments to target membranes. Studies on mutant and gene targeted mice suggest that Rab27a controls exocytosis of azurophil vesicles in neutrophils [78–80].

p14 deficiency was described by Bohn and colleagues in 2006 [81]. Four out of 15 offspring in the index family developed recurrent bronchopulmonary infections, hypopigmented skin, and neutropenia. The clinical phenotype of p14 deficiency was unique among the other described hypopigmentation-associated immunodeficiencies by causing short stature in the affected individuals. In vitro experiments showed impaired bactericidal activity and abnormal azurophil granules in p14 patient neutrophils. Furthermore, the distribution of the late endosomal compartment is perturbed in the absence of p14. The p14 protein is an adaptor of the MP1-MAPK scaffold complex and is involved in localization of MP1-MAPK to endosomes. The authors suggest that p14 is involved in granulocyte colony-stimulating factor (G-CSF) receptor signaling.

### 3.2. Mutations in Neutrophil Elastase and AP3

More than 50% of patients with congenital severe neutropenia and nearly all patients with cyclic neutropenia harbour mutations in the* ELANE* gene encoding for the neutrophil elastase (NE), a broad-specificity serine protease localized in azurophil granules [82–84]. The mechanism for how autosomal dominant mutations in* ELANE *induce neutropenia is still unclear [85]. The known human mutations do rarely affect protease activity of NE, nor its properties for substrate specificity [83]. Once produced, NE binds the adaptor protein 3 (AP3) and is shuttled from the trans-Golgi to azurophil granules. It is possible that* ELANE* mutations lead to mislocalization of NE within the cell or disturb NE protein folding [86]. Disruption of either NE or its cargo protein, the lysosomal transporter AP3 (encoded by* AP3B1*) [87, 88], perturbs the intracellular trafficking of NE to azurophil granules [89]. Moreover, mutated NE can induce the unfolded protein response in the endoplasmic reticulum [90, 91]. A recent report shows that certain patient mutations in* ELANE *force transcription to an alternative start site in the gene and production of an amino-terminal truncated form of NE that lack ER-localizing (pre) and zymogen-maintaining (pro) sequences yet retain essential catalytic residues [85]. The key role of* ELANE* in neutrophil homeostasis is also indicated by the development of SCN in patients carrying dominant negative mutations in the* GFI1 *gene,which is a transcriptional repressor of* ELANE *[92]. Although the mechanism for SCN induced by* ELANE *mutations is not directly linked to the actin cytoskeleton, it is likely that neutrophil deficiency that affects the actin cytoskeleton may have similar mislocalization of neutrophil proteases to vesicles and/or activation of the unfolded protein response.

### 3.3. Other Neutropenias with Vesicle Sorting Defects

Charcot-Marie-Tooth disease (CMT) is a progressive disorder of the peripheral nervous system and a genetic variant of CMT is caused by mutations in dynamin-2 (DNM2) [93]. DNM2 is a ubiquitously expressed mechanochemical protein with GTPase activity. DNM2 is associated with microtubules and is involved in endocytosis, cell motility, and centrosome organization. Several CMT patients with K558E and K558del DNM2 mutations have neutropenia [93]. The mechanism how DNM2 mutations cause neutropenia is unknown.

Cohen syndrome is a multiple congenital anomalies-mental retardation syndrome which is associated with neutropenia [94, 95]. No bone marrow morphological abnormalities were observed in Cohen syndrome patients; however their neutrophils exhibited greater adhesive capacity than the control ones and CD11b and CD62L surface expression was decreased on their neutrophils [96]. Cohen syndrome is caused by mutations in the vacuolar protein sorting 13B (*VPS13B*) gene [97]. Although the exact pathomechanism is unknown, vacuolar sorting proteins are involved in endosomal trafficking and protein recycling in the trans-Golgi network. Indicating their importance in granulocyte development, another VPS protein, VPS45 was recently found to be mutated in severe congenital neutropenia patients [98, 99]. In accordance with other severe congenital neutropenias, VPS45 mutant patients had severe infections and their neutrophils and bone marrow myeloid cells showed accelerated apoptosis. Peripheral neutrophils showed impaired migration and impaired superoxide production [98]. Vps45 is a member of the Sec1/Munc18 protein family that regulates the assembly of specific SNARE complexes. SNARE proteins mediate the fusion of lipid bilayers and serve a vital role in homeostasis of vesicle transport within the cell.

## 4. Other Neutrophil Deficiencies with Chemotaxis Involvement

Severe congenital neutropenia 4 (SCN4) is caused by homozygous mutations in the ubiquitously expressed catalytic subunit 3 of the glucose-6-phosphatase gene* (G6PC3)* [100]. Besides recurrent bacterial infections and neutropenia, SCN4 patients also show structural heart defects and urogenital abnormalities. Importantly, neutrophil development and function is also severely impaired in glycogen storage disease type Ib (GSD-Ib) which is caused by mutations in the glucose-6-phosphate transporter 1* (G6PT1)* [101, 102]. Chou and colleagues argue that a glucose-6-phosphatase complex which is composed of G6PC3 and G6PT1 is essential for neutrophil energy homeostasis and functionality by regulating endoplasmic reticulum glucose storage [103, 104]. Both G6PT and G6PC3 deficient neutrophils are impaired in chemotaxis, respiratory burst, and calcium mobilization [101, 102].

Papillon-Lefèvre syndrome (PLS) is characterized by palmoplantar keratosis and severe periodontitis which results in premature tooth loss [105]. PLS is caused by mutations in cathepsin C (CTSC) [105, 106], a lysosomal protease which is expressed highly in epithelial cells [106] and immune cells, including polymorphonuclear cells [107] and alveolar macrophages. In immune cells, cleavage by CTSC activates a variety of granule serine proteases by removing their inhibitory N-terminal dipeptides. Among others, CTSC targets are the neutrophil effectors NE, cathepsin G, and proteinase-3 [108, 109]. Increased susceptibility to infections in some cases [110] and neutrophil chemotaxis deficiency was reported in PLS patients [111]. It is controversial whether neutrophil chemotaxis is intrinsically defective in CTSC-deficient neutrophils. Based on the CTSC (also called dipeptidyl peptidase I; DPPI) knock-out mouse model, Adkison and colleagues argue that neutrophil-derived serine proteases are involved in the regulation of cytokine production at sites of inflammation [109].

Shwachman-Diamond syndrome (SDS) is characterized by pancreatic insufficiency, pancytopenia, and leukemia predisposition [112]. Bone marrow failure in patients with SDS is often manifested in neutropenia and peripheral SDS neutrophils are defective in chemotaxis towards fMLP [113, 114]. This disease is caused by mutations in the* SBDS* gene, encoding for a predicted RNA-processing protein, and suggests that SDS may be involved in RNA metabolism [115].

Even the most common genetic disease Chromosome 21 trisomy or Down syndrome causes a wide range of mild primary and secondary immunodeficiencies related to neutrophil dysfunction [116]. Trisomy 21 is characterized by high frequency of infections in the upper respiratory tract and periodontal disease which at least partially is attributed to reduced neutrophil chemotaxis [117].

## 5. Conclusion and Perspective

The dynamics of the actin cytoskeleton is a key feature of rapidly moving and acting cells such as neutrophils. A striking feature of neutrophil deficiency is that of all the hematopoietic cells, neutrophils are exceedingly vulnerable to loss of specific proteins or to changes in their activity. The reasons of this vulnerability perhaps originate from their unique developmental and functional requirements.

Neutrophils have a high turnover rate; they live for an average of 5 days in man [118] with a half-life of 7–10 hrs in human circulation [119]. A vast output of 10^11^ mature neutrophils/day from bone marrow requires efficient cell proliferation in the myeloid lineage, terminal differentiation, and egress from bone marrow. Defects in any of these processes cause SCN. An archetype of actin cytoskeleton disease that results in SCN is XLN, caused by overactivity of WASp. Given that all hematopoietic cells are dependent on WASp for their function it is reasonable to predict and evidence suggests that increased load of polymerized actin in XLN would affect the immune system broadly [18, 19, 21, 22]. However, the cardinal clinical feature of XLN patients is still neutropenia and neutrophil dysfunction. Our knowledge of the precise bone marrow pathology in XLN is limited due to few patients identified to date but it is likely that the fast dividing mitotic pool of granulocytic progenitor cells is highly sensitive to the increased cellular viscosity and aberrant cell division which is caused by an excess of cytoplasmic F-actin in XLN [19, 23].

Overactivity of the chemokine receptor CXCR4 in WHIM leads to an accumulation of neutrophils in the bone marrow. WHIM patient neutrophils adhere firmly to bone marrow stromal cells because of a failure to downregulate CXCR4 that is needed to egress from the bone marrow to the blood stream. In rats, mature neutrophils egress from the hematopoietic compartment to the circulation through the sinusoidal endothelium mostly via transcellular migration through tight-fitting pores which requires marked deformation of the neutrophil cell body [120]. To preserve their functional integrity, mature neutrophils are likely to require intact cytoskeletal regulation and vesicle structure when migrating through the sinusoidal endothelium in a narrow gap. These mechanical properties depend on the cortical F-actin content which differs between blood and bone marrow residing neutrophils [121].

The blood constantly flows past the tissues and neutrophils in the blood depend on integrin signaling for firm adhesion to the endothelial wall to reach an infected site. In order to efficiently migrate and become functionally highly active, neutrophils need to mobilize their secretory vesicles and upregulate CD11b [64]. This process is dependent on intact secretory pathways. Any defects in signaling of integrins are associated with severe neutropenia in LAD patients. You would predict that all hematopoietic cells that transmigrate to the tissue would be equally affected in LAD. However, unlike neutrophils, lymphocytes in CD11/CD18-deficient LAD patients are able to adhere to endothelial surfaces and emigrate to extravascular sites of inflammation. This adherence is probably mediated by the very late activation 4 (VLA-4) integrin receptors on lymphocytes, which bind to the vascular cell adhesion molecule 1 (VCAM-1) on the endothelial cells [122].

Inside the tissue, neutrophils are dependent on fast and dynamic migration to reach the microbes. Increased tension of the cell body would markedly reduce flexibility and can be caused by increased load of polymerized actin as proposed for XLN, decreased actin depolymerizing capacity in BRWS, or because of failure in vesicle fusion and fission as in CHS where neutrophils have accumulation of giant granules. Defects in the assembly of the NADPH complex due to mutations in NADPH subunits in CGD or in Rac2 deficiency ultimately leads to failure of microbial killing by neutrophils. Because neutrophils are packed with vesicles loaded with proteolytic enzymes and antimicrobial peptides, it is reasonable to predict that mislocalized packaging of proteins, such as implicated in cytosolic localization of NE in SCN, would be extremely harmful for the cell and lead to premature cell death. Future research will reveal if failure to regulate actin cytoskeleton dynamics for vesicle trafficking is a common feature in neutropenias caused by mutations in actin-regulating proteins such as Rac2, WASp, LSP1 in NAD 74/89, or in actin itself as in *β*actin deficiency. Moreover, the contribution of defects in microtubule organization and dynamics for vesicle trafficking in neutrophils remains to be determined.

Many attempts have been made to generate mouse models for human neutrophil dysfunctions. While some has been successful, including mice lacking NADPH subunits and Rac2 as a model for CGD and models for LADI–III [123], others have failed to induce neutrophil deficiency in mice. In one of the first attempts to generate a mouse model for the most common form of neutropenia, mice were gene-targeted to lack NE [124]. Given the severe effect of heterozygous* ELANE* mutations in SCN patients, the NE^−/−^ mice were surprisingly normal in terms of migration and killing of the Gram positive bacteria* Staphylococcus aureus *[124]. However, NE^−/−^ mice failed to kill Gram negative bacteria such as* Klebsiella pneumoniae* and* Escherichia coli *[124]. The reason that many mouse models may have a milder phenotype as compared to patients with similar mutation may be found in the species difference between mouse and man. Also, one confounding factor is that laboratory strains generally have low numbers of neutrophils [119, 125, 126]. Keeping this notion in mind, quite robust microbial challenges may be required to detect neutrophil deficiency in mice [123].

Despite some difficulties in generating valuable mouse models for human neutrophil deficiencies, animal models are superior when testing new treatment strategies and especially those with potential severe adverse risks for patients. Gene therapy is in the frontline for treatment of monogenetic diseases affecting the immune system. Gene therapy in two mouse models for CGD provided significant long-term correction of neutrophil function [127, 128]. However, several attempts worldwide have failed to provide long-term reconstitution of corrected neutrophils in CGD patients [129]. Gene therapy for Wiskott-Aldrich syndrome has been more satisfying with long-term engraftment of corrected cells and amelioration of disease [130]. Long-term treatment by GCSF, IFN*γ*, and high doses of antibiotics in neutrophil deficient patients are confounded by high risk to develop drug resistance and malignancies. Ongoing gene therapy trials worldwide give hope to diseases, including neutrophil deficiencies, where current treatment is unsatisfying.

## Figures and Tables

**Table 1:** Congenital defects in neutrophil dynamics.

Disease	Gene	Target	Neutropenia	Chemotaxis	Adhesion	Superoxide	Phagocytosis	Infections	Inheritance	Mutations	Other manifestations
A mutation of *β*-actin associated with neutrophil dysfunction	*β-Actin *	Cytoskeleton		+		+	+	+	AD	E364K	Thrombocytopenia, short stature, mental retardation
X-linked neutropenia	*WASp *		+		+	+	+	+	XL	L270P S272P I276S I294T	Lymphopenia
Neutrophil immunodeficiency syndrome	*Rac2 *			+	+	+	+	+	AD	D57N	
Neutrophil actin dysfunction (NAD) and NAD47/89	*?* *LSP1 *			+ +	+ +		+ +	+ +	AR AR		
Kostmann disease/ Severe congenital neutropenia 3 (SCN3)	*HAX1 *	(Cytoskeleton/ Apoptosis)	+	?				+	AR	W44X (72%) and other mutations	Neurological impairments

Leukocyte adhesion deficiency (LAD)	LAD I: *β*2 integrin family LAD II: *SLC35C1* LAD III: *FERMT3 *	Cell adhesion		+	+			+	AR	various	Leukocytosis LAD II: short stature, mental retardation LAD III: bleeding
WHIM syndrome	*CXCR4 *		+	+				+	AD	Truncations of C-terminus	B cell lymphopenia and hypo-gammaglobulinemia

Chédiak-Higashi syndrome	*LYST *	Vesicular transport, biogenesis, sorting	+	+				+	AR	various	Hypopigmentation, Neuropathies, immunodeficiency, hemophagocytic lymphohistiocytosis
Griscelli syndrome type 2	*RAB27A *		+					+	AR	various	Hypopigmentation, immunodeficiency, hemophagocytic lymphohistiocytosis
P14-deficiency	*MAPBPIP *		+					+	AR	3'UTR	Hypopigmentation, immunodeficiency, short stature
Hermansky-Pudlak syndrome type 2	*AP3B1 *		+					+	AR	various	Hypopigmentation, platelet, immunodeficiency
Charcot-Marie-Tooth disease, dominant intermediate B; (CMTDIB)	*DNM2 *		+						AD	various	Limb weakness and atrophy
Cohen syndrome	*VPS13B *		+		+			+	AR	various	Mental retardation, Microcephaly, hypotonia
VPS 45 mutation (SCN5)	*VPS45 *		+	+		+		+	AR	T224N E238K	Bone marrow fibrosis nephromegaly

Severe congenital neutropenia 1 (SCN1)	*ELANE *	Other	+					+	AD	various	
Severe congenital neutropenia 2 (SCN2)	*GFI1 *		+					+	AD	N382S K403R	Lymphopenia
Severe congenital neutropenia 4 (SCN4)	*G6PC3 *		+	+		+		+	AR	various	Heart defects, urogenital defects
Glycogen storage disease type 1b (GSD1B)	*G6PT1 *		+	+		+		+	AR	various	Impaired glucose homeostasis
Chronic granulomatous disease (CGD)	*gp*91^*phox*^ * gp*22^*phox*^ * p*47^*phox*^ * * *p*67^*phox*^ * * *p*40^*phox*^			+		+		+	XL AR AR AR AR	various	
Papillon-Lefèvre syndrome (PLS)	*CTSC *			+				+	AR	various	Hyperkeratosis Periodontitis
Shwachman-Diamond syndrome (SDS)	*SBDS *		+	+				+	AR	various	Pancreatic insufficiency, short stature, hematologic defects

AR: autosomal recessive, AD: autosomal dominant, XL: X-linked.

## Abbreviations

BRWS: Baraitser-Winter syndrome
CGD: Chronic granulomatous disease
CHS: Chédiak-Higashi syndrome
CMT: Charcot-Marie-Tooth disease
CTSC: Cathepsin C
fMLP: Formyl-methionyl-leucyl-phenylalanine
GCSF: Granulocyte colony-stimulating factor
ICAM: Intercellular adhesion molecule
LAD: Leukocyte adhesion deficiency
LSP1: Lymphocyte-specific protein 1
MTOC: Microtubule organizing center
NAD: Neutrophil actin dysfunction
NE: Neutrophil elastase
PLS: Papillon-Lefèvre syndrome
SCN: Severe congenital neutropenia
SDS: Shwachman-Diamond syndrome
WAVE: WASp-family verprolin-homologous protein
WAS: Wiskott-Aldrich syndrome
WASp: WAS protein
WHIM: Warts, hypogammaglobulinemia, infections, myelokathexis
XLN: X-linked neutropenia.

## References

[1] CEREDIH: The French PID study group (2010). The French national registry of primary immunodeficiency diseases. *Clinical Immunology*.

[2] Donadieu J, Fenneteau O, Beaupain B, Mahlaoui N, Chantelot CB (2011). Congenital neutropenia: diagnosis, molecular bases and patient management. *Orphanet Journal of Rare Diseases*.

[3] Pollard TD, Borisy GG (2003). Cellular motility driven by assembly and disassembly of actin filaments. *Cell*.

[4] Meili R, Firtel RA (2003). Two poles and a compass. *Cell*.

[5] Bunnell TM, Burbach BJ, Shimizu Y, Ervasti JM (2011). *β*-Actin specifically controls cell growth, migration, and the G-actin pool. *Molecular Biology of the Cell*.

[6] Shawlot W, Deng JM, Fohn LE, Behringer RR (1998). Restricted *β*-galactosidase expression of a hygromycin-lacZ gene targeted to the *β*-actin locus and embryonic lethality of *β*-actin mutant mice. *Transgenic Research*.

[7] Nunoi H, Yamazaki T, Tsuchiya H (1999). A heterozygous mutation of *β*-actin associated with neutrophil dysfunction and recurrent infection. *Proceedings of the National Academy of Sciences of the United States of America*.

[8] Procaccio V, Salazar G, Ono S (2006). A mutation of *β*-actin that alters depolymerization dynamics is associated with autosomal dominant developmental malformations, deafness, and dystonia. *The American Journal of Human Genetics*.

[9] Rivière JB, van Bon BW, Hoischen A (2012). *De novo* mutations in the actin genes *ACTB* and *ACTG1* cause Baraitser-Winter syndrome. *Nature Genetics*.

[10] di Donato N, Rump A, Koenig R (2014). Severe forms of Baraitser-Winter syndrome are caused by ACTB mutations rather than ACTG1 mutations. *European Journal of Human Genetics*.

[11] Johnston JJ, Wen KK, Keppler-Noreuil K (2013). Functional analysis of a de novo ACTB mutation in a patient with atypical Baraitser-Winter syndrome. *Human Mutation*.

[12] Moulding DA, Record J, Malinova D, Thrasher AJ (2013). Actin cytoskeletal defects in immunodeficiency. *Immunological Reviews*.

[13] Massaad MJ, Ramesh N, Geha RS (2013). Wiskott-Aldrich syndrome: a comprehensive review. *Annals of the New York Academy of Sciences*.

[14] Zhang H, Schaff UY, Green CE (2006). Impaired integrin-dependent function in Wiskott-Aldrich syndrome protein-deficient murine and human neutrophils. *Immunity*.

[15] Snapper SB, Meelu P, Nguyen D (2005). WASP deficiency leads to global defects of directed leukocyte migration in vitro and in vivo. *Journal of Leukocyte Biology*.

[16] Kumar S, Xu J, Perkins C (2012). Cdc42 regulates neutrophil migration via crosstalk between WASp, CD11b, and microtubules. *Blood*.

[17] Devriendt K, Kim AS, Mathijs G (2001). Constitutively activating mutation in WASP causes X-linked severe congenital neutropenia. *Nature Genetics*.

[18] Ancliff PJ, Blundell MP, Cory GO (2006). Two novel activating mutations in the Wiskott-Aldrich syndrome protein result in congenital neutropenia. *Blood*.

[19] Moulding DA, Blundell MP, Spiller DG (2007). Unregulated actin polymerization by WASp causes defects of mitosis and cytokinesis in X-linked neutropenia. *The Journal of Experimental Medicine*.

[20] Beel K, Cotter MM, Blatny J (2009). A large kindred with X-linked neutropenia with an I294T mutation of the Wiskott-Aldrich syndrome gene. *British Journal of Haematology*.

[21] Burns SO, Killock DJ, Moulding DA (2010). A congenital activating mutant of WASp causes altered plasma membrane topography and adhesion under flow in lymphocytes. *Blood*.

[22] Westerberg LS, Meelu P, Baptista M (2010). Activating WASP mutations associated with X-linked neutropenia result in enhanced actin polymerization, altered cytoskeletal responses, and genomic instability in lymphocytes. *Journal of Experimental Medicine*.

[23] Moulding DA, Moeendarbary E, Valon L, Record J, Charras GT, Thrasher AJ (2012). Excess F-actin mechanically impedes mitosis leading to cytokinesis failure in X-linked neutropenia by exceeding Aurora B kinase error correction capacity. *Blood*.

[24] Houk AR, Jilkine A, Mejean CO (2012). Membrane tension maintains cell polarity by confining signals to the leading edge during neutrophil migration. *Cell*.

[25] Roberts AW, Kim C, Zhen L (1999). Deficiency of the hematopoietic cell-specific Rho family GTPase Rac2 is characterized by abnormalities in neutrophil function and host defense. *Immunity*.

[26] Aspenstrom P (2014). BAR domain proteins regulate Rho GTPase signaling. *Small GTPases*.

[27] Dinauer MC (2003). Regulation of neutrophil function by Rac GTPases. *Current Opinion in Hematology*.

[28] Ambruso DR, Knall C, Abell AN (2000). Human neutrophil immunodeficiency syndrome is associated with an inhibitory Rac2 mutation. *Proceedings of the National Academy of Sciences of the United States of America*.

[29] Williams DA, Tao W, Yang F (2000). Dominant negative mutation of the hematopoietic-specific Rho GTPase, Rac2, is associated with a human phagocyte immunodeficiency. *Blood*.

[30] Accetta D, Syverson G, Bonacci B (2011). Human phagocyte defect caused by a Rac2 mutation detected by means of neonatal screening for T-cell lymphopenia. *The Journal of Allergy and Clinical Immunology*.

[31] Dinauer MC (2005). Chronic granulomatous disease and other disorders of phagocyte function. *Hematology/the Education Program of the American Society of Hematology: American Society of Hematology: Education Program*.

[32] Kim JS, Huang TY, Bokoch GM (2009). Reactive oxygen species regulate a slingshot-cofilin activation pathway. *Molecular Biology of the Cell*.

[33] van Rheenen J, Condeelis J, Glogauer M (2009). A common cofilin activity cycle in invasive tumor cells and inflammatory cells. *Journal of Cell Science*.

[34] Nimnual AS, Taylor LJ, Bar-Sagi D (2003). Redox-dependent downregulation of Rho by Rac. *Nature Cell Biology*.

[35] Reeves EP, Lu H, Jacobs HL (2002). Killing activity of neutrophils is mediated through activation of proteases by K+ flux. *Nature*.

[36] Boxer LA, Hedley Whyte ET, Stossel TP (1974). Neutrophil actin dysfunction and abnormal neutrophil behavior. *The New England Journal of Medicine*.

[37] Southwick FS, Dabiri GA, Stossel TP (1988). Neutrophil actin dysfunction is a genetic disorder associated with partial impairment of neutrophil actin assembly in three family members. *The Journal of Clinical Investigation*.

[38] Southwick FS, Howard TH, Holbrook T, Anderson DC, Stossel TP, Arnaout MA (1989). The relationship between CR3 deficiency and neutrophil actin assembly. *Blood*.

[39] Coates TD, Torkildson JC, Torres M, Church JA, Howard TH (1991). An inherited defect of neutrophil motility and microfilamentous cytoskeleton associated with abnormalities in 47-Kd and 89-Kd proteins. *Blood*.

[40] Howard T, Li Y, Torres M, Guerrero A, Coates T (1994). The 47-kD protein increased in neutrophil actin dysfunction with 47- and 89-kD protein abnormalities is lymphocyte-specific protein. *Blood*.

[41] Li Y, Guerrero A, Howard TH (1995). The actin-binding protein, lymphocyte-specific protein 1, is expressed in human leukocytes and human myeloid and lymphoid cell lines. *Journal of Immunology*.

[42] Howard TH, Hartwig J, Cunningham C (1998). Lymphocyte-specific protein 1 expression in eukaryotic cells reproduces the morphologic and motile abnormality of NAD 47/89 neutrophils. *Blood*.

[43] Jongstra-Bilen J, Misener VL, Wang C (2000). LSP1 modulates leukocyte populations in resting and inflamed peritoneum. *Blood*.

[44] van de Vijver E, van den Berg TK, Kuijpers TW (2013). Leukocyte adhesion deficiencies. *Hematology/Oncology Clinics of North America*.

[45] Delon I, Brown NH (2007). Integrins and the actin cytoskeleton. *Current Opinion in Cell Biology*.

[46] DeMali KA, Wennerberg K, Burridge K (2003). Integrin signaling to the actin cytoskeleton. *Current Opinion in Cell Biology*.

[47] Kinashi T (2005). Intracellular signalling controlling integrin activation in lymphocytes. *Nature Reviews Immunology*.

[48] Hauck F, Klein C (2013). Pathogenic mechanisms and clinical implications of congenital neutropenia syndromes. *Current Opinion in Allergy and Clinical Immunology*.

[49] Klein C, Grudzien M, Appaswamy G (2007). HAX1 deficiency causes autosomal recessive severe congenital neutropenia (Kostmann disease). *Nature Genetics*.

[50] Suzuki Y, Demoliere C, Kitamura D, Takeshita H, Deuschle U, Watanabe T (1997). HAX-1, a novel intracellular protein, localized on Mitochondria, directly associates with HS1, a substrate of Src family Tyrosine kinases. *Journal of Immunology*.

[51] Cilenti L, Soundarapandian MM, Kyriazis GA (2004). Regulation of HAX-1 anti-apoptotic protein by Omi/HtrA2 protease during cell death. *The Journal of Biological Chemistry*.

[52] Sharp TV, Wang HW, Koumi A (2002). K15 protein of Kaposi’s sarcoma-associated herpesvirus is latently expressed and binds to HAX-1, a protein with antiapoptotic function. *Journal of Virology*.

[53] Gallagher AR, Cedzich A, Gretz N, Somlo S, Witzgall R (2000). The polycystic kidney disease protein PKD2 interacts with Hax-1, a protein associated with the actin cytoskeleton. *Proceedings of the National Academy of Sciences of the United States of America*.

[54] Ramsay AG, Keppler MD, Jazayeri M (2007). HS1-associated protein X-1 regulates carcinoma cell migration and invasion via clathrin-mediated endocytosis of integrin *α*v*β*6. *Cancer Research*.

[55] Radhika V, Onesime D, Ha JH, Dhanasekaran N (2004). G*α*13 stimulates cell migration through cortactin-interacting protein Hax-1. *The Journal of Biological Chemistry*.

[56] Cavnar PJ, Berthier E, Beebe DJ, Huttenlocher A (2011). Hax1 regulates neutrophil adhesion and motility through RhoA. *Journal of Cell Biology*.

[57] Hernandez PA, Gorlin RJ, Lukens JN (2003). Mutations in the chemokine receptor gene CXCR4 are associated with WHIM syndrome, a combined immunodeficiency disease. *Nature Genetics*.

[58] Martin C, Burdon PCE, Bridger G, Gutierrez-Ramos J, Williams TJ, Rankin SM (2003). Chemokines acting via CXCR2 and CXCR4 control the release of neutrophils from the bone marrow and their return following senescence. *Immunity*.

[59] Suratt BT, Petty JM, Young SK (2004). Role of the CXCR4/SDF-1 chemokine axis in circulating neutrophil homeostasis. *Blood*.

[60] Kawai T, Malech HL (2009). WHIM syndrome: congenital immune deficiency disease. *Current Opinion in Hematology*.

[61] Balabanian K, Lagane B, Pablos JL (2005). WHIM syndromes with different genetic anomalies are accounted for by impaired CXCR4 desensitization to CXCL12. *Blood*.

[62] Kawai T, Choi U, Cardwell L (2007). WHIM syndrome myelokathexis reproduced in the NOD/SCID mouse xenotransplant model engrafted with healthy human stem cells transduced with C-terminus-truncated CXCR4. *Blood*.

[63] Walters KB, Green JM, Surfus JC, Yoo SK, Huttenlocher A (2010). Live imaging of neutrophil motility in a zebrafish model of WHIM syndrome. *Blood*.

[64] Borregaard N, Cowland JB (1997). Granules of the human neutrophilic polymorphonuclear leukocyte. *Blood*.

[65] Aspenström P (2008). Roles of F-BAR/PCH proteins in the regulation of membrane dynamics and actin reorganization. *International Review of Cell and Molecular Biology*.

[66] Aspenström P, Lindberg U, Hall A (1996). Two GTPases, Cdc42 and Rac, bind directly to a protein implicated in the immunodeficiency disorder Wiskott-Aldrich syndrome. *Current Biology*.

[67] Rohatgi R, Ma L, Miki H (1999). The interaction between N-WASP and the Arp2/3 complex links Cdc42- dependent signals to actin assembly. *Cell*.

[68] Stinchcombe J, Bossi G, Giffiths GM (2004). Linking albinism and immunity: the secrets of secretory lysosomes. *Science*.

[69] Kaplan J, De Domenico I, Ward DM (2008). Chediak-Higashi syndrome. *Current Opinion in Hematology*.

[70] Blume RS, Bennett JM, Yankee RA, Wolff SM (1968). Defective granulocyte regulation in the Chediak-Higashi syndrome. *The New England Journal of Medicine*.

[71] Clark RA, Kimball HR (1971). Defective granulocyte chemotaxis in the Chediak-Higashi syndrome. *Journal of Clinical Investigation*.

[72] Root RK, Rosenthal AS, Balestra DJ (1972). Abnormal bactericidal, metabolic, and lysosomal functions of Chediak-Higashi Syndrome leukocytes. *The Journal of Clinical Investigation*.

[73] Ganz T, Metcalf JA, Gallin JI, Boxer LA, Lehrer RI (1988). Microbicidal/cytotoxic proteins of neutrophils are deficient in two disorders: Chediak-Higashi syndrome and “specific” granule deficiency. *Journal of Clinical Investigation*.

[74] Kjeldsen L, Calafat J, Borregaard N (1998). Giant granules of neutrophils in Chediak-Higashi syndrome are derived from azurophil granules but not from specific and gelatinase granules. *Journal of Leukocyte Biology*.

[75] Barbosa MDFS, Nguyen QA, Tchernev VT (1996). Identification of the homologous beige and Chediak-Higashi syndrome genes. *Nature*.

[76] Griscelli C, Durandy A, Guy-Grand D, Daguillard F, Herzog C, Prunieras MA (1978). A syndrome associating partial albinism and immunodeficiency. *The American Journal of Medicine*.

[77] Ménasché G, Pastural E, Feldmann J (2000). Mutations in RAB27A cause Griscelli syndrome associated with haemophagocytic syndrome. *Nature Genetics*.

[78] Johnson JL, Brzezinska AA, Tolmachova T (2010). Rab27a and Rab27b regulate neutrophil azurophilic granule exocytosis and NADPH oxidase activity by independent mechanisms. *Traffic*.

[79] Singh RK, Liao W, Tracey-White D (2012). Rab27a-mediated protease release regulates neutrophil recruitment by allowing uropod detachment. *Journal of Cell Science*.

[80] Munafó DB, Johnson JL, Ellis BA, Rutschmann S, Beutler B, Catz SD (2007). Rab27a is a key component of the secretory machinery of azurophilic granules in granulocytes. *The Biochemical Journal*.

[81] Bohn G, Allroth A, Brandes G (2007). A novel human primary immunodeficiency syndrome caused by deficiency of the endosomal adaptor protein p14. *Nature Medicine*.

[82] Horwitz M, Benson KF, Person RE, Aprikyan AG, Dale DC (1999). Mutations in ELA2, encoding neutrophil elastase, define a 21-day biological clock in cyclic haematopoiesis. *Nature Genetics*.

[83] Horwitz MS, Duan Z, Korkmaz B, Lee H, Mealiffe ME, Salipante SJ (2007). Neutrophil elastase in cyclic and severe congenital neutropenia. *Blood*.

[84] Dale DC, Person RE, Bolyard AA (2000). Mutations in the gene encoding neutrophil elastase in congenital and cyclic neutropenia. *Blood*.

[85] Tidwell T, Wechsler J, Nayak RC (2014). Neutropenia-associated ELANE mutations disrupting translation initiation produce novel neutrophil elastase isoforms. *Blood*.

[86] Xia J, Link DC (2008). Severe congenital neutropenia and the unfolded protein response. *Current Opinion in Hematology*.

[87] Shotelersuk V, Dell'Angelica EC, Hartnell L, Bonifacino JS, Gahl WA (2000). A new variant of Hermansky-Pudlak syndrome due to mutations in a gene responsible for vesicle formation. *The American Journal of Medicine*.

[88] Dell'Angelica EC, Shotelersuk V, Aguilar RC, Gahl WA, Bonifacino JS (1999). Altered trafficking of lysosomal proteins in Hermansky-Pudlak syndrome due to mutations in the *β*3A subunit of the AP-3 adaptor. *Molecular Cell*.

[89] Benson KF, Li F, Person RE (2003). Mutations associated with neutropenia in dogs and humans disrupt intracellular transport of neutrophil elastase. *Nature Genetics*.

[90] Köllner I, Sodeik B, Schreek S (2006). Mutations in neutrophil elastase causing congenital neutropenia lead to cytoplasmic protein accumulation and induction of the unfolded protein response. *Blood*.

[91] Grenda DS, Murakami M, Ghatak J (2007). Mutations of the ELA2 gene found in patients with severe congenital neutropenia induce the unfolded protein response and cellular apoptosis. *Blood*.

[92] Person RE, Li F, Duan Z (2003). Mutations in proto-oncogene GFI1 cause human neutropenia and target ELA2. *Nature Genetics*.

[93] Züchner S, Noureddine M, Kennerson M (2005). Mutations in the pleckstrin homology domain of dynamin 2 cause dominant intermediate Charcot-Marie-Tooth disease. *Nature Genetics*.

[94] Cohen MM, Hall BD, Smith DW, Graham CB, Lampert KJ (1973). A new syndrome with hypotonia, obesity, mental deficiency, and facial, oral, ocular, and limb anomalies. *The Journal of Pediatrics*.

[95] Kivitie-Kallio S, Rajantie J, Juvonen E, Norio R (1997). Granulocytopenia in Cohen syndrome. *British Journal of Haematology*.

[96] Olivieri O, Lombardi S, Russo C, Corrocher R (1998). Increased neutrophil adhesive capability in Cohen syndrome, an autosomal recessive disorder associated with granulocytopenia. *Haematologica*.

[97] Kolehmainen J, Black GCM, Saarinen A (2003). Cohen syndrome is caused by mutations in a novel gene, COH1, encoding a transmembrane protein with a presumed role in vesicle-mediated sorting and intracellular protein transport. *The American Journal of Human Genetics*.

[98] Vilboux T, Lev A, Malicdan MCV (2013). A congenital neutrophil defect syndrome associated with mutations in VPS45. *The New England Journal of Medicine*.

[99] Stepensky P, Saada A, Cowan M (2013). The Thr224Asn mutation in the VPS45 gene is associated with the congenital neutropenia and primary myelofibrosis of infancy.. *Blood*.

[100] Boztug K, Appaswamy G, Ashikov A (2009). A syndrome with congenital neutropenia and mutations in G6PC3. *The New England Journal of Medicine*.

[101] Gerin I, Veiga-da-Cunha M, Achouri Y, Collet JF, van Schaftingen E (1997). Sequence of a putative glucose 6-phosphate translocase, mutated in glycogen storage disease type Ib. *The FEBS Letters*.

[102] Narisawa K, Igarashi Y, Otomo H, Tada K (1978). A new varient of glycogen storage disease Type I probably due to a defect in the glucose-6-phosphate transport system. *Biochemical and Biophysical Research Communications*.

[103] Cheung YY, Kim SY, Yiu WH (2007). Impaired neutrophil activity and increased susceptibility to bacterial infection in mice lacking glucose-6-phosphatase-*β*. *The Journal of Clinical Investigation*.

[104] Jun HS, Weinstein DA, Lee YM, Mansfield BC, Chou JY (2014). Molecular mechanisms of neutrophil dysfunction in glycogen storage disease type Ib. *Blood*.

[105] Toomes C, James J, Wood AJ (1999). Loss-of-function mutations in the cathepsin C gene result in periodontal disease and palmoplantar keratosis. *Nature Genetics*.

[106] Hart TC, Hart PS, Bowden DW (1999). Mutations of the cathepsin C gene are responsible for Papillon-Lefevre syndrome. *Journal of Medical Genetics*.

[107] Rao NV, Rao GV, Hoidal JR (1997). Human dipeptidyl-peptidase I: gene characterization, localization, and expression. *The Journal of Biological Chemistry*.

[108] McGuire MJ, Lipsky PE, Thiele DL (1993). Generation of active myeloid and lymphoid granule serine proteases requires processing by the granule thiol protease dipeptidyl peptidase I. *Journal of Biological Chemistry*.

[109] Adkison AM, Raptis SZ, Kelley DG, Pham CTN (2002). Dipeptidyl peptidase I activates neutrophil-derived serine proteases and regulates the development of acute experimental arthritis. *The Journal of Clinical Investigation*.

[110] Haneke E (1979). The Papillon-Lefevre syndrome: keratosis palmoplantaris with periodontopathy. Report of a case and review of the cases in the literature. *Human Genetics*.

[111] Firatli E, Tüzün B, Efeoğlu A (1996). Papillon-Lefèvre syndrome: analysis of neutrophil chemotaxis. *Journal of Periodontology*.

[112] Smith OP (2002). Shwachman-Diamond syndrome. *Seminars in Hematology*.

[113] Stepanovic V, Wessels D, Goldman FD, Geiger J, Soll DR (2004). The chemotaxis defect of Shwachman-Diamond Syndrome leukocytes. *Cell Motility and the Cytoskeleton*.

[114] Orelio C, Kuijpers TW (2009). Shwachman-diamond syndrome neutrophils have altered chemoattractant-induced F-actin polymerization and polarization characteristics. *Haematologica*.

[115] Boocock GRB, Morrison JA, Popovic M (2003). Mutations in SBDS are associated with Shwachman-Diamond syndrome. *Nature Genetics*.

[116] Ram G, Chinen J (2011). Infections and immunodeficiency in Down syndrome. *Clinical and Experimental Immunology*.

[117] Novo E, Garcia MI, Lavergne J (1993). Nonspecific immunity in Down syndrome: a study of chemotaxis, phagocytosis, oxidative metabolism, and cell surface marker expression of polymorphonuclear cells. *The American Journal of Medical Genetics*.

[118] Pillay J, den Braber I, Vrisekoop N (2010). In vivo labeling with ^2^H_2_O reveals a human neutrophil lifespan of 5.4 days. *Blood*.

[119] von Vietinghoff S, Ley K (2008). Homeostatic regulation of blood neutrophil counts. *The Journal of Immunology*.

[120] Burdon PCE, Martin C, Rankin SM (2008). Migration across the sinusoidal endothelium regulates neutrophil mobilization in response to ELR + CXC chemokines. *British Journal of Haematology*.

[121] Saito H, Lai J, Rogers R, Doerschuk CM (2002). Mechanical properties of rat bone marrow and circulating neutrophils and their responses to inflammatory mediators. *Blood*.

[122] Schwartz BR, Wayner EA, Carlos TM, Ochs HD, Harlan JM (1990). Identification of surface proteins mediating adherence of CD11/CD18-deficient lymphoblastoid cells to cultured human endothelium. *Journal of Clinical Investigation*.

[123] Schäffer AA, Klein C (2013). Animal models of human granulocyte diseases. *Hematology/Oncology Clinics of North America*.

[124] Belaaouaj A, Mccarthy R, Baumann M (1998). Mice lacking neutrophil elastase reveal impaired host defense against gram negative bacterial sepsis. *Nature Medicine*.

[125] TheJacksonLaboratory Hematological survey of 11 inbred strains of mice. http://www.phenome.jax.org/.

[126] Wakeman L, Al-Ismail S, Benton A (2007). Robust, routine haematology reference ranges for healthy adults. *International Journal of Laboratory Hematology*.

[127] Björgvinsdóttir H, Ding C, Pech N, Gifford MA, Li LL, Dinauer MC (1997). Retroviral-mediated gene transfer of gp91^*phox*^ into bone marrow cells rescues defect in host defense against *Aspergillus fumigatus* in murine X- linked chronic granulomatous disease. *Blood*.

[128] Mardiney M, Jackson SH, Spratt SK, Li F, Holland SM, Malech HL (1997). Enhanced host defense after gene transfer in the murine p47(phox)- deficient model of chronic granulomatous disease. *Blood*.

[129] Mukherjee S, Thrasher AJ (2013). Gene therapy for PIDs: progress, pitfalls and prospects. *Gene*.

[130] Griffith LM, Cowan MJ, Notarangelo LD (2014). Primary Immune Deficiency Treatment Consortium (PIDTC) report. *The Journal of Allergy and Clinical Immunology*.

